# Malaria in pregnant women in an area with sustained high coverage of insecticide-treated bed nets

**DOI:** 10.1186/1475-2875-7-133

**Published:** 2008-07-21

**Authors:** Abdunoor M Kabanywanyi, John R MacArthur, Wilma A Stolk, J Dik F Habbema, Hassan Mshinda, Peter B Bloland, Salim Abdulla, S Patrick Kachur

**Affiliations:** 1 Ifakara Health Research and Development Centre, Tanzania; 2 Centers for Disease Control and Prevention, USA; 3 Department of Public Health, Erasmus MC, University Medical Center Rotterdam, The Netherlands

## Abstract

**Background:**

Since 2000, the World Health Organization has recommended a package of interventions to prevent malaria during pregnancy and its sequelae that includes the promotion of insecticide-treated bed nets (ITNs), intermittent preventive treatment in pregnancy (IPTp), and effective case management of malarial illness. It is recommended that pregnant women in malaria-endemic areas receive at least two doses of sulphadoxine-pyrimethamine in the second and third trimesters of pregnancy. This study assessed the prevalence of placental malaria at delivery in women during 1^st ^or 2^nd ^pregnancy, who did not receive intermittent preventive treatment for malaria (IPTp) in a malaria-endemic area with high bed net coverage.

**Methods:**

A hospital-based cross-sectional study was done in Ifakara, Tanzania, where bed net coverage is high. Primi- and secundigravid women, who presented to the labour ward and who reported not using IPTp were included in the study. Self-report data were collected by questionnaire; whereas neonatal birth weight and placenta parasitaemia were measured directly at the time of delivery.

**Results:**

Overall, 413 pregnant women were enrolled of which 91% reported to have slept under a bed net at home the previous night, 43% reported history of fever and 62% were primigravid. Malaria parasites were detected in 8% of the placenta samples; the geometric mean (95%CI) placental parasite density was 3,457 (1,060–11,271) parasites/μl in primigravid women and 2,178 (881–5,383) parasites/μl in secundigravid women. Fifteen percent of newborns weighed <2,500 g at delivery. Self-reported bed net use was statistically associated with lower risk for low birth weight [OR 0.34 (95% CI: 0.16–0.74) and OR 0.22 (95% CI: 0.08–0.59) for untreated and treated bed nets, respectively], but was not associated with placental parasitaemia [OR 0.74 (0.21–2.68) and OR 1.64 (0.44–6.19) for untreated and treated bed nets, respectively].

**Conclusion:**

The observed incidence of LBW and prevalence of placental parasitaemia at delivery suggests that malaria remains a problem in pregnancy in this area with high bed net coverage when eligible women do not receive IPTp. Delivery of IPTp should be emphasized at all levels of implementation to achieve maximum community coverage.

## Background

Malaria infection during pregnancy poses substantial risks to the mother, her fetus and the newborn. Consequences of malaria infection during pregnancy include severe anemia, placental parasitaemia and intra-uterine growth retardation. These factors contribute to low birth weight (LBW), one of the principal causes of infant mortality in the African region[1]. Primigravid, in particular, and secundigravid women are at higher risk for placental malaria than women with multiple prior pregnancies[2].

Since 2000, the World Health Organization (WHO) has recommended a package of interventions to prevent malaria during pregnancy and its sequelae[3]. This includes the promotion of ITNs, IPTp, and effective case management of malarial illness. With respect to IPTp, WHO recommends that pregnant women in malaria-endemic areas receive at least two doses of sulphadoxine-pyrimethamine in the second and third trimesters of pregnancy[4]. Many countries have adopted the WHO recommendations, but implementation has often been hampered by constraints within health service delivery system or individual perceptions of IPTp[5,6]. These factors include health service delivery systems burdened by poor drug supply, poor health worker practices and low attendance or late presentation to antenatal clinics.

The National Malaria Control Programme (NMCP) for mainland Tanzania adopted the WHO recommendations for prevention of malaria in pregnancy in 2001. In the same year, the national first line antimalarial treatment drug was changed from chloroquine to a single dose of sulphadoxine-pyrimethamine and this also became the first choice drug for IPTp [7]. In 2004, during a health facility survey in 21 districts across mainland Tanzania the NMCP discovered that nearly 60% of pregnant women received at least one dose of IPTp [8].

Since the mid-1990s, the Tanzania Ministry of Health endorsed in its national guidelines the use of ITNs as a household malaria prevention tool. Various non-governmental organizations and projects have since promoted subsidized ITN sales in the country under the direction of the Ministry. In communities surrounding Ifakara town, ITN promotion efforts have been underway for more than a decade now. Net coverage surveys in the region in 1995 and 1996 revealed that 17% and 37% of households, respectively, owned at least one bed net [9,10]. This proportion increased markedly after intensive promotion of highly subsidized ITNs. In 2004, it was recorded that more than 80% of households in villages surrounding Ifakara owned a bed net and/or reported using bed nets. Moreover, the current district wide bed net coverage including an area of moderate ITN promotion activities stands at 78.4%[11]. The increased bed net coverage made an important contribution to prevention of malaria illness episodes among children less than five years[12]. The impact on other risk groups has not been studied in detail. Whereas the impact on other groups besides children has been under reported, this study investigated the occurrence of malaria in pregnant women who did not report receiving IPTp during their pregnancy. Placental parasitaemia and neonatal birth weight were used as outcome indicators. In particular, the study investigated the extent to which use of bed nets contributed to protecting this group of women from the impact of malaria in pregnancy.

## Materials and methods

### Study area and population

The study area included Ifakara town and the surrounding villages in the rice growing plains of the Kilombero River Valley. Monsoon tropical rains fall from December to May leading to an average annual rainfall of 1,200 mm. Malaria transmission ranges from intense to moderate and transmission is perennial, peaking after the period of long rains[13,14]. A hospital-based study was carried out at St. Francis Designated District Hospital (SFDDH) in Ifakara town, Tanzania. Ifakara town is the administrative headquarters for Kilombero District which covers 14,900 km^2 ^and contains more than 50 villages. SFDDH is a 400-bed hospital with the labour ward providing services to pregnant women from Ifakara (about 75% of attendees) and from the surrounding villages (about 15% of attendees), belonging to both Kilombero District and neighboring Ulanga District. The remaining 10% of pregnant women come from other parts of Kilombero and Ulanga Districts as well as other neighbouring districts within Morogoro Region.

The population of Ifakara is about 100,000 people and the 25 villages surrounding Ifakara have a combined population of another 90,000 who participate in a continuous Demographic Surveillance System (DSS). Women of reproductive age in the DSS account for 20% of the general population whereby 3% of them bear a child each year.

### Recruitment procedures

A hospital based cross-sectional study included all pregnant women who gave consent to be interviewed during labour. The one-year enrollment period started in December 2003 through to December 2004. A trained nurse midwife interviewed all eligible women who presented for delivery at the labour ward, using a structured questionnaire, documented delivery outcomes, and collected placenta samples after delivery. A woman was eligible if she was in her 1st or 2nd pregnancy and had not received IPTp from an antenatal care provider during their recently completed pregnancy. Pregnant women who were seriously ill at the time of delivery were excluded from the study. This study occurred alongside a randomized controlled trial of three alternative regimens for IPTp which will be reported later.

### Data and sample collection

Care and admission record taking for all pregnant women during delivery followed the normal hospital routine. During each interview, the nurse midwife counterchecked the information on drug use from the antenatal clinic (ANC) cards and case management logs. In the questionnaire, information about the place of domicile, fever episodes, treatment of fever including antimalarial drug use, and the use of bed nets during the woman's recently completed pregnancy was obtained. After delivery, a trained nurse midwife scored the baby (Apgar score) at one minute and five minutes later, measured the birth weight and gestational age of the baby, and collected two blood slides from the placenta. Thick and thin blood films were prepared from samples collected using a round ended plastic spatulae from incised maternal and cord parts of the placenta. A research microscopist air-dried and stained slides (one for back up purposes) with 10% Giemsa for 30 minutes and a study clinician randomly selected 10% of all slides to be quality controled by a 2^nd ^reader. *Plasmodium falciparum *malaria parasitaemia per μl was determined by counting parasites per 200 leukocytes, assuming 8,000 leukocytes per μl. The results were made available to all mothers before they were discharged from the hospital for feedback purposes.

### Data-analysis procedures

Data entry was done using FoxPro^® ^database software version 7 (Microsoft Corporation, Redmond USA 2001) at the Ifakara Health Research and Development Centre (IHRDC). Data cleaning and analysis were done using Stata version 8.0 (Stata Corporation, College Station, Texas USA) software. Newborn birth weight was classified as normal (>= 2,500 g) or low (<2,500 g)[15]. Univariate analysis was used to identify predictors of placental parasitaemia and low birth weight. Age, sex, fever, use of antimalarial drugs and management of fever, use of mosquito bed nets, parity, and patient home were each examined in univariate analyses. Those predictors that were significant at the p = 0.10 level were subsequently included in a multivariate logistic regression model. In the multivariate model, an interaction term was introduced for combinations of variables that demonstrated a relevant relationship with each outcome of interest. The presence of statistical interaction in the model was tested using a likelihood ratio test (LRT).

### Ethical consideration

This study was approved as part of the larger clinical trial by the institutional review boards of the Centers for Disease Control and Prevention, IHRDC, and the Tanzanian Medical Research Coordinating Committee. Eligible women provided written informed consent before being enrolled.

## Results

Four hundred and thirteen pregnant women were enrolled into the study upon presenting for delivery in the labour ward. Characteristics of these women and their newborns are given in Table 1. Primigravid women accounted for 62% of those enrolled in the study. Three quarters of all women were living in Ifakara or villages surrounding the town. Overall net coverage was very high with 91% of women reporting that they had slept under a bed net at home the night prior to admission to the hospital for delivery. Of these women, 23% reported that their net was treated with insecticide within the previous year. Of the 416 infants born to 413 enrolled women, 408 (98%) were live singleton deliveries, 6 (1.4%) were live twin infants and 2 (0.5%) were stillborn. The overall mean birth weight among singleton babies was 2,881 g. The mean birth weight of the singleton newborns was 2,793 g (SD,423 g) and 3,023 g (SD,444 g) among primigavid and secundigravid respectively. The mean birth weight of singleton babies born to primigravid was significantly lower than that of those born to secundigravid women (p < 0.01). Sixty infants (15%) among the live singleton births were classified as low birth weight.

**Table 1:** Maternal and infant's characteristics

Attribute	Overall
Age mean (SD), years	20.3 (3.6)

Patient's village of residence:	
Ifakara & its vicinity No. (%)	307 (74.3)
Beyond Ifakara region No. (%)	106 (25.7)

Reported to have slept under bed-net at home the night prior to admission for delivery:	
Untreated No. (%)	280 (67.8)
Treated No. (%)	96 (23.2)
Total No. (%)	376 (91.0)

Reported fever episode No. (%)	178 (43)

With placenta parasitaemia No. (%)	33 (8)

Geometric (95% CI) mean placenta parasitaemia:	
Primigravid mean parasites/μl	3,457 (1,060–11,271)
Secundigravid mean parasites/μl	2,178 (881–5,383)

Total number of newborn:	416
Alive singletons No. (%)	408 (98.1)
Alive twins No. (%)	6 (1.4)
Stillbirths No. (%)	2 (0.5)

Neonatal birth weight:	
No. (singleton including stillbirths)	404
Birth weight mean (SD), g	2,881 (445)

Neonates sex	
Male No. (%)	213 (51.2)
Female No. (%)	203 (48.8)

Incidence of LBW:	
Birth weight <2500 g No. (%)	60 (14.9)
Male	29 (48.3)
Female	31 (51.7)

Overall, 43% of the mothers reported to have experienced at least one episode of illness with fever during the course of pregnancy and 33 (8%) of the placental samples were positive for *P. falciparum *(Table 1). The geometric mean placental parasite density was 3,457 (1,060–11,271) in primigravid women, and 2,178 (881–5,383) [95% Confidence intervals, CI] parasites/μl in secundigravid women. Among the women who reported fever (n = 178), only 58% reported self use of anti-malarial medications whereas the remaining used antipyretics and other forms of drugs including antibiotics (Table 2).

**Table 2:** Risk factors for placental parasitaemia at delivery

Variable	At risk of placenta parasitaemia (n)	Univariate analysis odds ratio (95% CI)	LR (p-value)	Multivariate analysis odds ratio (95% CI)
Age	413	1.08 (0.99 – 1.19)	0.09	1.07 (0.97 – 1.17)

Patient's home:			0.31	
Ifakara & vicinity	307	1		
Beyond Ifakara	106	1.50 (0.70 – 3.21)		

Parity:			0.20	
Secundigravid	157	1		
Primigravid	256	0.63 (0.31 – 1.28)		

Use of bednet:			0.09	
Never used	37	1		
Untreated	280	0.73 (0.2 – 2.6)		0.74 (0.21 – 2.68)
Treated bednet	96	1.78 (0.5 – 6.6)		1.64 (0.44 – 6.19)

Report of self-medication:			0.28	
Never used	236	1		
Antimalarial	103	1.41 (0.59 – 3.34)		
Other forms	74	2.04 (0.85 – 4.88)		

Reported fever episode:			0.31	
Never reported	235	1		
Reported	178	1.45 (0.71 – 2.95)		

Incidence of birth weight	(n = 404)		0.31	
Normal BW	344	1		
LBW	60	1.62 (0.67 – 3.91)		

¶-N = 33 cases of placenta parasitaemia

LR-likelihood ratio

In univariate analyses, mother's age and the use of a bed net were associated with placenta parasitaemia at delivery (*p *= 0.09 for both) (Table 2). In the multivariate logistic regression analysis however, neither age nor bed net use was significantly associated with placental parasitaemia (LR, *p *= 0.08).

The risk of LBW was related to the mother's age (*p *< 0.01), parity (*p *= 0.04) and the use of a bed net (*p *= 0.05) (Table 3) in univariate analyses. Only the use of a bed net remained significant in the multivariate model (*p *< 0.01). There was not any evidence of interaction between age and parity in the multivariate model that included an interaction term (age times parity) [LRT, *p *= 0.07]. Bed net users had a considerably lower risk of delivering an infant with LBW than non-users. This protective effect was slightly stronger for women who used treated bed nets (OR 0.22, CI: 0.08 – 0.59) than for women who used untreated nets (OR 0.34, CI: 0.16 – 0.74).

**Table 3:** Risk factors for low birth weight

Variable	At risk of LBW (n)	Univariate analysis odds ratio (95% (CI)	LR (*p*-value)	Multivariate analysis odds ratio (95% (CI)	LRT
Age	404	0.89 (0.81 – 0.97)	< 0.01	0.92 (0.83 – 1.02)	

Patient's home:			0.66		
Ifakara & vicinity	299	1			
Beyond Ifakara	105	1.15 (0.62 – 2.12)			

Parity:			0.04		
Secundigravid	156	1			
Primigravid	248	1.89 (1.02 – 3.48)		1.40 (0.69 – 2.82)	

Age*Parity (interaction)	404			0.82 (0.67 – 1.01)	0.07

Use of bednet:			0.05		
Never used	37	1			
Untreated	273	0.36 (0.17 – 0.77)		0.34 (0.16 – 0.74)	
Treated bednet	94	0.19 (0.07 – 0.53)		0.22 (0.08 – 0.59)	

Report of self-medication:			0.65		
Never used	228	1			
Antimalarial	103	0.98 (0.52 – 1.86)			
Other forms	73	0.66 (0.29 – 1.48)			

Reported fever episode:			0.78		
Never reported	229	1			
Reported	175	1.08 (0.62 – 1.88)			

Placenta parasitaemia:			0.31		
Absent	371	1			
Present	33	1.62 (0.67 – 3.91)			

Neonates sex			0.24		
Male	213	1			
Female	203	1.15 (0.66 – 1.98)			

¶-N = 60 cases of LBW (Birth weight < 2500 g)

LR-likelihood ratio

LRT-Likelihood ratio test for interaction

## Discussion

This study showed that in the absence of effective IPTp coverage some risk from malaria during pregnancy persisted. Interestingly, despite the absence of IPTp, the prevalence of placental parasitaemia among primigravid and secundigravid women in Ifakara has remained relatively low. Reasons for this low prevelance may include the substantial increase in ITN coverage over the past 10 years and the general ecological trend previously described in Ifakara town[14]. Since the inception of the Kilombero Valley social marketing programme for Insecticide-Treated Bed Nets (KINET), ITN coverage in the area has increased from 37% in 1996 to over 85% in 2005. Previous studies conducted in the same facility in 1994–95 on malaria and pregnancy revealed placental parasitaemia rates in women of all parity categories at 35.2% even though some of the study population used chloroquine for prophylaxis [15].

It can be argued that the true prevalence of placental parasitaemia may have been somewhat higher, because the microscopic detection has limited sensitivity compared to the more sensitive and more expensive approach of placental histopathology[16]. In this study, the microscopic placental examination allowed for a simpler and quicker analysis that provided evidence on the presence of placental malaria in women who did not receive IPTp.

This study recorded a 15% incidence of LBW and the risk of delivering a LBW infant was lower in mothers who used a bed net (either treated or untreated). Moreover, the study did not find any effect of bed net use on the risk of placental parasitaemia; quite possibly due to the small number of cases found. In addition, this study found no direct evidence that the reduced risk of LBW in bed net-users was directly attributable to the prevention of placental malaria. This observation may also be related to low power of the study (few mothers had placental parasitaemia) and the fact that placental malaria infections earlier in pregnancy may have been cleared by the time of delivery. Although factors such as socio-economic (SES) or nutritional status could have contributed to the risk for LBW, this study did not assess the contribution of those factors.

This study reported high occurrence of bed net use among pregnant women, which may suggest a strong awareness of malaria in pregnancy in this specific group. In western Kenya, for instance, it was demonstrated that women who thought malaria in pregnancy caused foetal problems were more likely to have used an ITN[17]. The use of mosquito bed nets in pregnancy in Ifakara reflects the high level coverage achieved after many years of an intense social marketing programme and the availability of highly subsidized ITNs since 1996[10]. These results support the efforts of the malaria in pregnancy community to advocate for full implementation of IPTp, even in the face of other malaria control interventions.

## Conclusion

The observed incidence of LBW and prevalence of placental parasitaemia at delivery suggest that malaria remains a problem in pregnancy, even in areas with high bed net coverage for women who do not receive IPTp. Delivery of IPTp should be emphasized at all levels of implementation to achieve maximum community coverage.

## Authors' contributions

AM was responsible for the protocol development, study design, patients recruitment, data analysis and developing the manuscript. JM was responsible for the protocol development, study design, patients recruitment and for comments on the early version of the manuscript. WS was responsible for data interpretation and developing the manuscript. JDH was responsible for data interpretation and manuscript development. HM was responsible for the study design and comments on the early version of the manuscript. SA was responsible for the protocol development, study design, recruitment set up, data interpretation and developing of the manuscript. SPK was responsible for the protocol development, study design and developing of the manuscript.
